# Effects of Pressure Support Ventilation Mode on Emergence Time and Intra-Operative Ventilatory Function: A Randomized Controlled Trial

**DOI:** 10.1371/journal.pone.0115139

**Published:** 2014-12-23

**Authors:** Xavier Capdevila, Boris Jung, Nathalie Bernard, Christophe Dadure, Philippe Biboulet, Samir Jaber

**Affiliations:** 1 Anaesthesia and Critical Care Department A, Lapeyronie Teaching Hospital, Centre Hospitalier Universitaire Montpellier, Unité INSERM U1046, Montpellier, 34295, France; 2 Anaesthesiology and Intensive Care, Anaesthesia and Critical Care Department B, Saint Eloi Teaching Hospital, Unité INSERM U1046, Université Montpellier 1, Centre Hospitalier Universitaire Montpellier, Montpellier, 34295, France; San Raffaele Scientific Institute, Italy

## Abstract

We tested the hypothesis that pressure-support ventilation (PSV) allows a reduction in emergence time and laryngeal mask airway (LMA) removal time after general anesthesia compared to volume-controlled mechanical ventilation (CMV). Because spontaneous breathing (SB) is often used with LMA under general anesthesia, patients were allocated randomly to three groups (CMV, SB and PSV). Thirty-six consecutive ASA I–II patients scheduled for knee arthroscopic surgery under general anesthesia with a LMA and breathing throughout the ventilator circuit were included. Hemodynamic and ventilatory variables were recorded before and 10-min after general anesthesia-induction, at the surgical incision, at the end of anaesthetic drugs infusion and when the patient was totally awake (which defines emergence time). LMA removal time, drug consumption were recorded at the end of the surgical procedure. Leak fraction around the LMA was also evaluated. LMA removal time was significantly higher in the CMV-group (18±6 min) compared to both SB (8±4 min) and PSV (7±4 min, *P*<0.05) groups as well as for emergence time: CMV-group (32±12 min), SB (17±7 min) and PSV (13±6 min, *P*<0.05) groups. Total propofol consumption was significantly lower in the PSV-group (610±180 mg) than in both CMV (852±330 mg) and SB (734±246 mg, *P<0.05*) groups. Air leaks around the LMA was significantly higher in the CMV-group than in the SB and PSV groups (16% vs 3% and 7%, all *P*<0.05). In conclusion, in knee arthroscopic surgery, in comparison to CMV, PSV use during general anesthesia in unparalyzed patients decreases LMA removal time, propofol consumption and leaks around LMA while improving ventilatory variables without adverse effects.

**Trial Registration:**

Controlled-Trials.com ISRCTN17382426

## Introduction

Pressure-support ventilation (PSV) is a mode of spontaneous ventilation which has been used for a long time in critical care but has only recently been introduced to general anaesthetic practice [1], [2]. PSV mode was proposed to allow the patient to take spontaneous breaths, when appropriate, without “fighting the ventilator” [3]–[5]. Contrary to pressure-controlled ventilation which generates a decelerating flow, but with a constant inspiratory time, in PSV mode the patient imposes his or her respiratory rate and inspiratory time [5]–[7]. One of the potential advantages of PSV is a better patient-ventilator synchrony and the associated decrease in work of breathing and improved breathing comfort [4], [8]. That's why PSV was used to enable a smooth transition between apnoea and spontaneous ventilation in anaesthesia practices. PSV has also been widely used as partial ventilatory support to improve gas exchange as compared to Volume Controlled Mechanical Ventilation (CMV) [9], [10]. Recently, many manufacturers have integrated PSV into anaesthesia ventilators [11]–[13]. The increased use of the laryngeal mask airway [14] has encouraged clinicians to allow patients to breathe spontaneously. The use of PSV in the operating room was reported in anesthetized patients with laryngeal mask airway (LMA) [15], [16]. Gas exchange during general anesthesia is accomplished with CMV [1]. To our knowledge, there are no previous intra-operative studies comparing the CMV and PSV modes under general anesthesia with LMA. However, spontaneous breathing (SB) is often used with LMA, but may provide less effective gas exchange than CMV [5], [17]. Indeed, hypercapnic acidosis and an increased work of breathing often occur during SB under general anaesthesia both in healthy and non-healthy patients [18]. We then designed a randomized study comparing CMV to PSV under general anesthesia with LMA. Because, SB is a popular ventilatory mode used under LMA, an additional group of patients under SB was also evaluated. In the present randomized study, we tested the hypothesis that PSV allows a reduction of LMA removal and emergence time associated to anaesthetic drugs consumption compared to CMV.

## Materials and Methods

The study was approved by the Lapeyronie University Hospital ethics committee. Informed written consent was obtained from all patients. The protocol for this trial and supporting CONSORT checklist are available as supporting information; see S1 Checklist and S1 Protocol [19]. ASA physical status I and II patients scheduled for elective knee arthroscopic surgery under general anesthesia with a classic LMA and mechanical ventilation were included in this study. Exclusion criteria were emergency procedures, ASA physical status >II and patient refusal. Premedication consisted in oral hydroxyzine (1 mg kg^−1^) and a gel-capsule of methylen blue (2 mg). In operating room (OR), the patients were allocated randomly into three groups (SB, CMV and PSV) using a simple randomization without blocking. The investigators generated a random-number table on a computer (function RAND in Excel software), used the table to prepare envelopes for random patient allocation. General anesthesia was induced using propofol 2.5 mg kg^−1^, fentanyl 2 µg kg^−1^ and was maintained with propofol 0.05 mg kg.^−1^ min^−1^ and fentanyl 0.02 µg kg^−1^ min^−1^ with nitrous oxide in oxygen, with a fraction of inspired oxygen of 0.4.

LMA (size 4, females; size 5, males) were inserted according to the manufacturer's instructions (Laryngeal MaskCo., Henley-on-Thames, UK). The volume of air in the cuff was adjusted at an airway sealing pressure greater than 15 cmH_2_O. Mechanical ventilation was initiated (Servo 900C ventilator Siemens-Elema, Solna, Sweden). In SB-group, patients underwent SB throughout the ventilator circuit, in CMV-group, patients underwent CMV (expiratory tidal volume: VTe of 8 mL/kg, a respiratory rate (RR) of 10 cycles/min and an inspiratory/total duty cycle (Ti/Ttot) ratio of 1∶3 with a time plateau pressure at 15% of the inspiratory time) and in PSV-group patients underwent PSV (PSV level was set to obtain a VTe between 7–8 mL/kg and RR between 10–16 cycles/min, inspiratory trigger was fixed at −2 cmH_2_O and the termination of inspiratory support was fixed at the drop of flow at 25% peak inspiratory flow value). Ventilation was performed with zero positive end expiratory pressure in all three groups.

**Figure 1:**
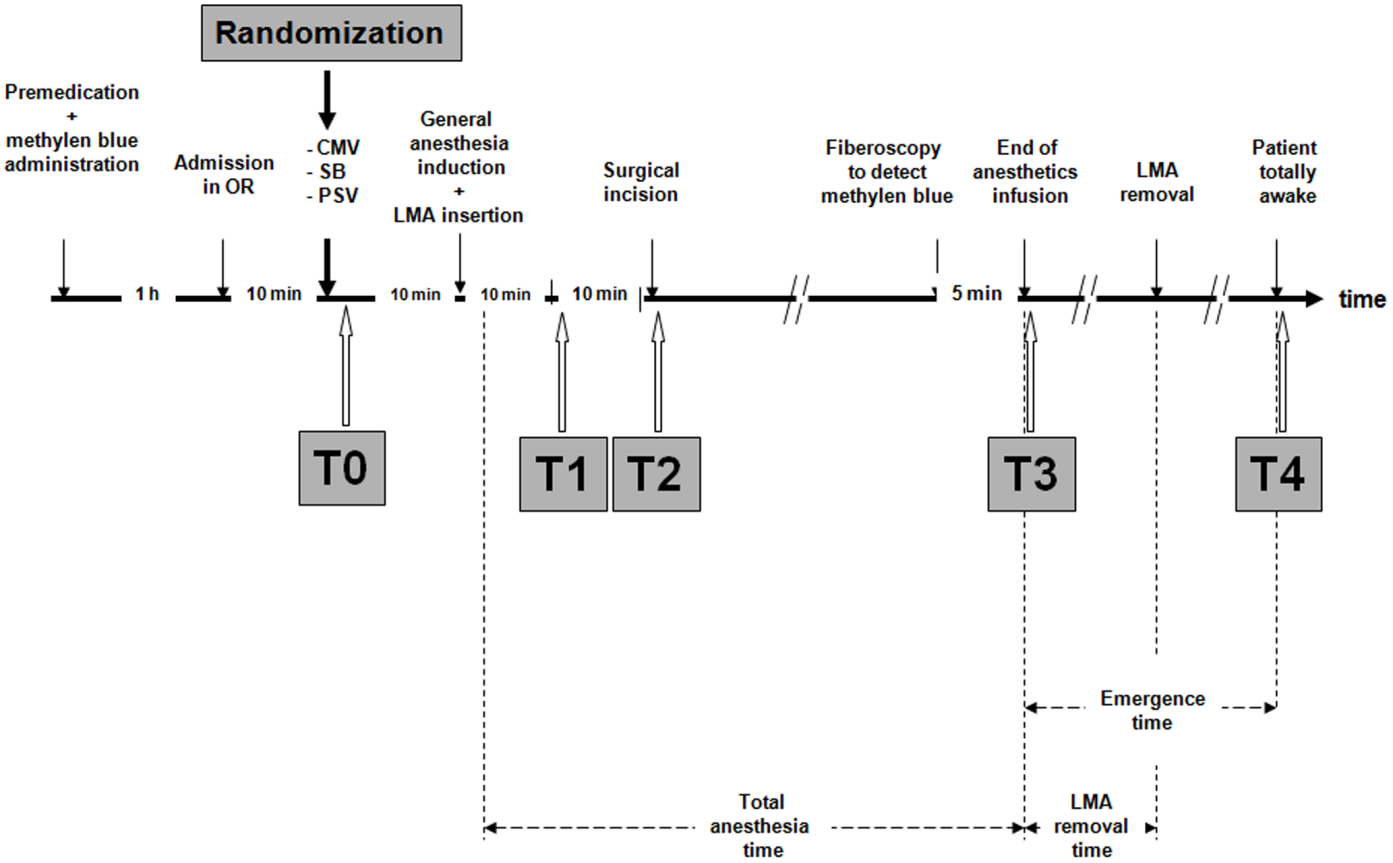
Schematic illustration of the study design. Patient totally awake was defined as a patient who obtained a 10 point score on the five questions test (see text).

Monitoring included electrocardiography, mean arterial blood pressure (MAP) (monitored noninvasively), pulsoxymetry (Sp_O2_) and end expiratory concentrations of oxygen, carbon dioxide (Pet_CO2_), and nitrous oxide (Capnomac Ultima; Datex-Ohmeda, Helsinki, Finland). The Capnomac Ultima (Datex-Ohmeda, Helsinki, Finland) was also used to continuously monitor the airway pressures and flow waveforms which allowed detection of the wasted inspiratory effort on the expiratory part of the flow waveform [7], [20]. The anaesthetic regimen was individually adapted to hemodynamics variables [21]. Additional boluses of propofol 0.25 mg/kg and fentanyl 25 µg were given in case of a 20 beat min^−1^ increase in heart rate and/or a 25% increase in MAP during 2 min. The study design is summarized in Fig. 1. All data were recorded at baseline (T0: before general anesthesia induction), 10 min after general anesthesia induction (T1), at the surgical incision (T2), at the end of anaesthetic drugs infusion (T3) and when the patient was totally awake (T4). Drugs consumption during general anesthesia and duration of general anesthesia were recorded at the end of the surgical procedure. We also calculated drugs consumption during general anesthesia (except induction dose) normalized to weight and duration of general anesthesia expressed in mg kg^−1^ min^−1^ for propofol and µg kg^−1^ min^−1^ for fentanyl. Hemodynamic (Heart rate, MAP) and ventilatory variables (RR, VTi, VTe, Ti/Ttot, maximal and plateau pressures, Sp_O2_, Pet_CO2_) were recorded during a 2-min stable state for each period. Peak pressure, plateau pressure and flow were measured with Capnomac Ultima at the tube opening. Airway leaks were determined by noting the difference between inspired and expired tidal volume (VTi-VTe). Airway occlusion pressure (P0.1) was measured at each point except in T1, T2 and T3 for the CMV group [22]. For T0 and T4, ventilatory variables were obtained in awake patients spontaneously breathing through the circuit via a mouth piece with a nose clip.

Although the staff members who collected data during surgery were aware of the group assignments, end points assessors were unaware of these assignments throughout the study. At the end of surgery, airway fiberoscopy was performed throughout the LMA to detect the presence or absence of methylene blue regurgitation [23]. The removal time (open mouth to command) of the LMA was determined by questioning every minute the patient and the emergence time was defined as the time to obtain a 10 point score on a five questions test. Each of the following items; 1) month of birth; 2) date of surgery; 3) day of the week; 4) address of the patient; 5) simple addition, was scored 0 (no response), 1 (inexplicit response) or 2 (good response).

### Statistical analysis

The primary end point was LMA removal time with PSV in comparison to CMV. The secondary end points were LMA removal time with SB in comparison to PSV and CMV; respiratory variables and total anaesthetic drugs consumption between the three groups. Previous studies have shown that general anesthesia emergence time with LMA removal in CMV patients ranges from 15–20 min, with an average of 18 min and an SD of 6 min. We postulated a difference of 50% in the LMA removal time in the PSV group [24]. Therefore, taking into account that we evaluated three groups and accepting a type I error risk of 2.5% per comparison (5% overall) and a type II error risk of 20% (power 80%), 10 patients would be required in each group to evaluate our hypothesis. To take in account potential missing data, 12 patients were included in each group. Data are expressed as mean ± SD. Comparison of two means was performed using the Student t test after checking that the distribution was normal. Comparison of several means was performed using repeated-measures analysis of variance and the Newman-Keuls test. *P<*0.05 was taken as significant. Statistical analysis were performed using NCSS 6.0 software (Statistical Solutions Ltd., Cork, Ireland).

## Results

**Figure 2:**
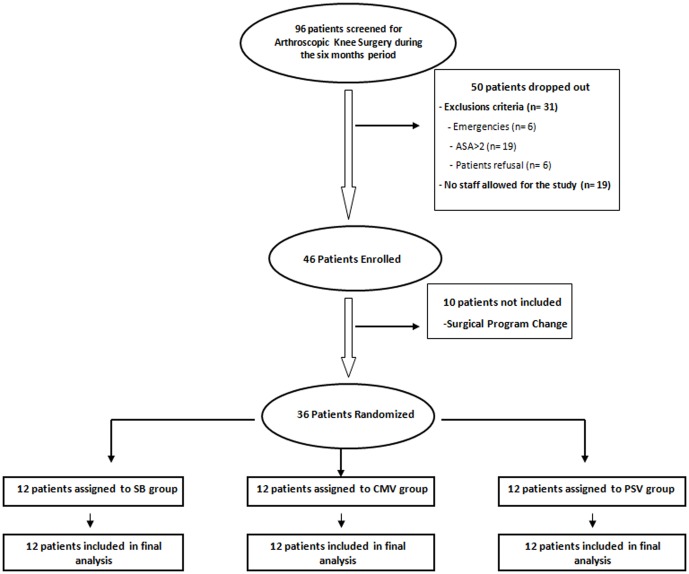
Completed CONSORT flowchart of the study.

Thirty-six consecutive patients were included and allocated randomly to the three groups of 12 patients (CMV, SB and PSV). A completed CONSORT flowchart of the study is reported in Fig. 2. No patient was dropped out. No significant difference was observed between the three groups for patient characteristics and total anaesthesia duration (Table 1). One patient in the CMV-group had the presence of methylene blue in the LMA, without airway complication.

**Table 1:** Patient and anesthetic characteristics.

	CMV (n = 12)	SB (n = 12)	PSV (n = 12)
**Age (years)**	33±12	29±6	31±9
**Sex (M/F)**	7/5	7/5	6/6
**Height (cm)**	173±9	173±7	172±11
**Weight (kg)**	67±11	70±12	70±13
**Total anesthesia time (min)**	81±21	85±20	75±18

Data are expressed as mean ± SD

No significant difference was observed between the three groups for patient characteristics and general anesthesia duration.

***Abbreviations:*** CMV: controlled mechanical ventilation; SB: spontaneous breathing; PSV: Pressure Support Ventilation.

**Figure 3:**
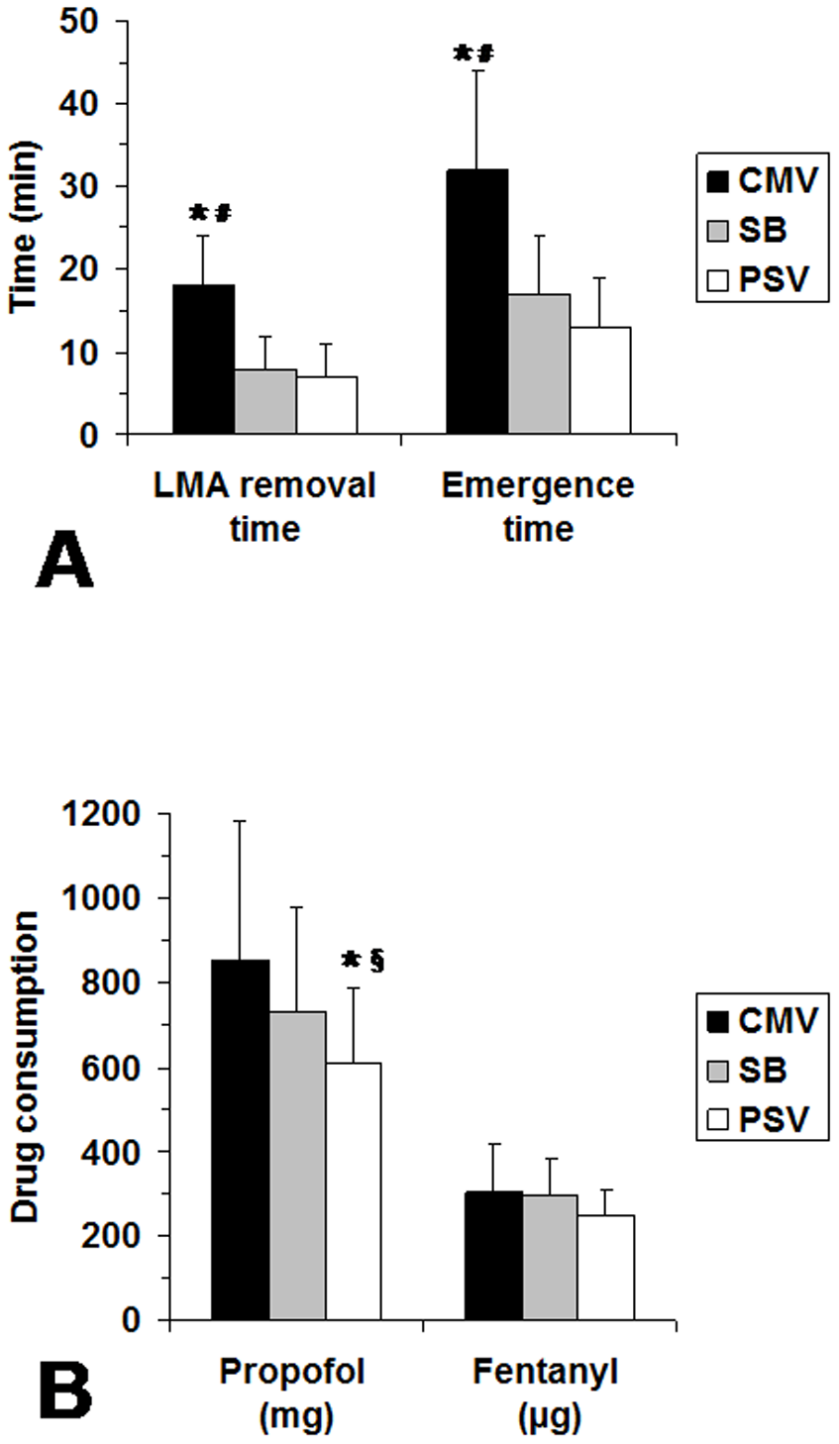
Laryngeal mask airway removal, emergence times and total anaesthesia drug consumptions between the three groups. (*A*) Comparisons of the laryngeal mask airway removal and emergence times between the three groups: CMV: controlled mechanical ventilation; SB: spontaneous breathing and PSV: Pressure Support Ventilation. (*B*) Comparisons of the total anaesthesia drug consumptions between the three groups (CMV, SB and PSV). Results are expressed as mean ± SD (n = 12 in each group). * *P*<0.05 CMV vs PSV; # *P*<0.05 CMV vs SB; § *P*<0.05 SB vs PSV.

LMA removal and emergence times were significantly longer in the CMV-group in comparison to PSV group (Fig. 3A). Similarly, LMA removal and emergence times were significantly longer in the CMV-group in comparison to SB group (Fig. 3A). Total propofol consumption was significantly lower in the PSV-group than in both CMV and SB groups (Fig. 3B). Normalized drugs consumption values were significantly higher in the CMV group than in both SB and PSV groups for propofol (0.13 vs 0.09 vs 0.08 mg kg^−1^ min^−1^ respectively, *P*<0.05) and not significantly different between groups for fentanyl (0.031 vs 0.026 vs 0.021 µg kg^−1^ min^−1^ respectively).

Respiratory and hemodynamic data are reported in Table 2. For SB, VT significantly decreased, but remained constant for both CMV and PSV throughout the study period (Table 2). For SB, P0.1 increased significantly from baseline period to the end of anaesthetic drugs infusion, whereas it remained below 1.5 cmH_2_O for all study measurements in the PSV-group (Table 2).

**Table 2:** Respiratory and hemodynamic variables during the five periods of the study for the three groups.

		T0 (baseline)	T1 (after induction)	T2 (surgical incision)	T3 (end of general anesthesia infusion)	T4 (awake)
RR (cycles/min)	CMV	12±2	10±0	10±0	10±0	12±3
	SB	13±3	11±3	11±2	11±2	13±3
	PSV	13±2	11±1	11±2	11±1	13±2
						
VTexp (L/min)	CMV	740±200	610±130	590±80	600±90	710±180
	SB	620±120	380±100^#^ § £	400±70^#^ § £	420±120^#^ § £	600±200
	PSV	650±160	630±140	630±140	640±130	650±140
						
Ti/Ttot	CMV	0.41±0.04	0.41±0.05* ^#^	0.39±0.05* ^#^	0.39±0.05* ^#^	0.38±0.05
	SB	0.40±0.03	0.28±0.05£	0.26±0.04£	0.28±0.05£	0.38±0.05
	PSV	0.37±0.05	0.29±0.04£	0.28±0.02£	0.28±0.03£	0.37±0.04
						
Plateau pressure (cmH_2_O)	CMV	-	11±3	12±3	12±3	-
	SB	-	-	-	-	-
	PSV	-	9±2	10±2	10±2	-
						
P0.1 (cmH_2_O)	CMV	1.2±0.4	-	-	-	1.4±0.5
	SB	1.3±0.5	1.4±0.7	1.8±0.9£	2.0±0.6£	1.4±0.5
	PSV	1.4±0.8	1.0±0.5	1.0±0.4§	1.0±0.6§	1.3±0.7
						
Sp_O2_ (%)	CMV	98.1±0.7	98.2±0.7	98.0±0.9	98.3±0.8	98.1±1.0
	SB	98.3±0.8	98.1±0.7	98.1±0.5	98.1±0.9	98.0±0.9
	PSV	97.4±0.6	98.9±0.6	98.9±0.7	98.8±0.8	97.9±0.7
						
Heart rate (beat min^−1^)	CMV	79±8	61±10£	61±8£	63±9£	76±12
	SB	72±8	62±5£	61±4£	65±7£	72±13
	PSV	75±7	60±8£	55±7£	55±7£	69±7
						
MAP (mmHg)	CMV	92±10	75±11£	90±18	92±16	93±11
	SB	90±13	78±14£	81±11	89±17	90±13
	PSV	92±11	76±11£	87±9	91±9	89±10
						

Data are expressed as mean ± SD.

***Abbreviations:*** CMV: controlled mechanical ventilation; SB: spontaneous brpoeathing; PSV: Pressure Support Ventilation; RR: respiratory rate; VTexp: expiratory tidal volume; Ti/Ttot: duty cycle; P0.1: occlusion pressure at 100 ms; SpO2: oxygen saturation.

MAP: mean arterial pressure.

^*^ *P*<0.05 CMV vs PSV.

# *P*<0.05 CMV vs SB.

§ *P*<0.05 SB vs PSV.

£ comparison to T0 (i.e, T1 vs T0; T2 vs T0; T3 vs T0).

**Figure 4:**
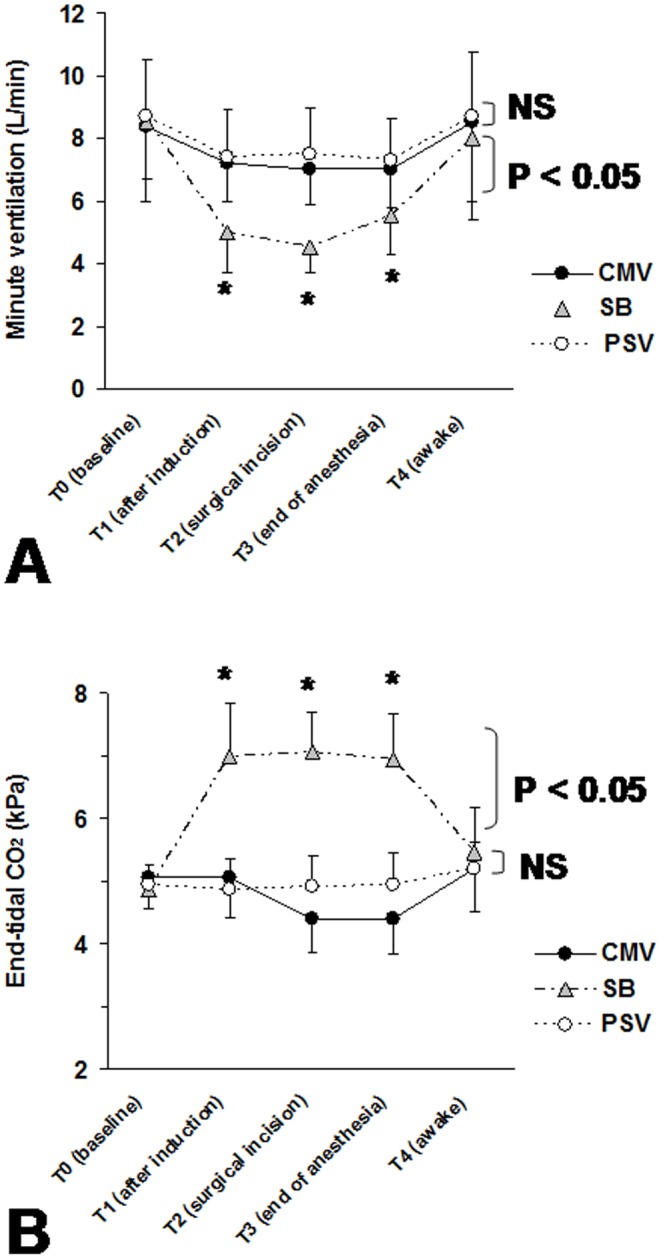
Minute ventilation and end-tidal CO2 during the study periods. (*A*) minute ventilation and (*B*) end-tidal CO2 (Pet_CO2_) at baseline (T0: before general anesthesia induction), 10 min after general anesthesia induction (T1), at the surgical incision (T2), at the end of anaesthetic drugs infusion (T3) and when the patient was totally awake (T4) obtained for the three groups: CMV-group (Controlled Mechanical Ventilation), SB-group (Spontaneous Breathing) and PSV-group (Pressure Support Ventilation). Data are expressed as mean ± SD. *P* value refers to between-groups comparison. *P<0.05 versus baseline values, NS: not significant.

Fig. 4 shows the evolution of minute ventilation and Pet_CO2_ values. For the SB-group, the significant decrease of minute ventilation (Fig. 4A) was associated with a significant increase in Pet_CO2_ (Fig. 4B) for periods T1, T2 and T3 in comparison to periods T0 (baseline) and T4 (baseline-return).

**Figure 5:**
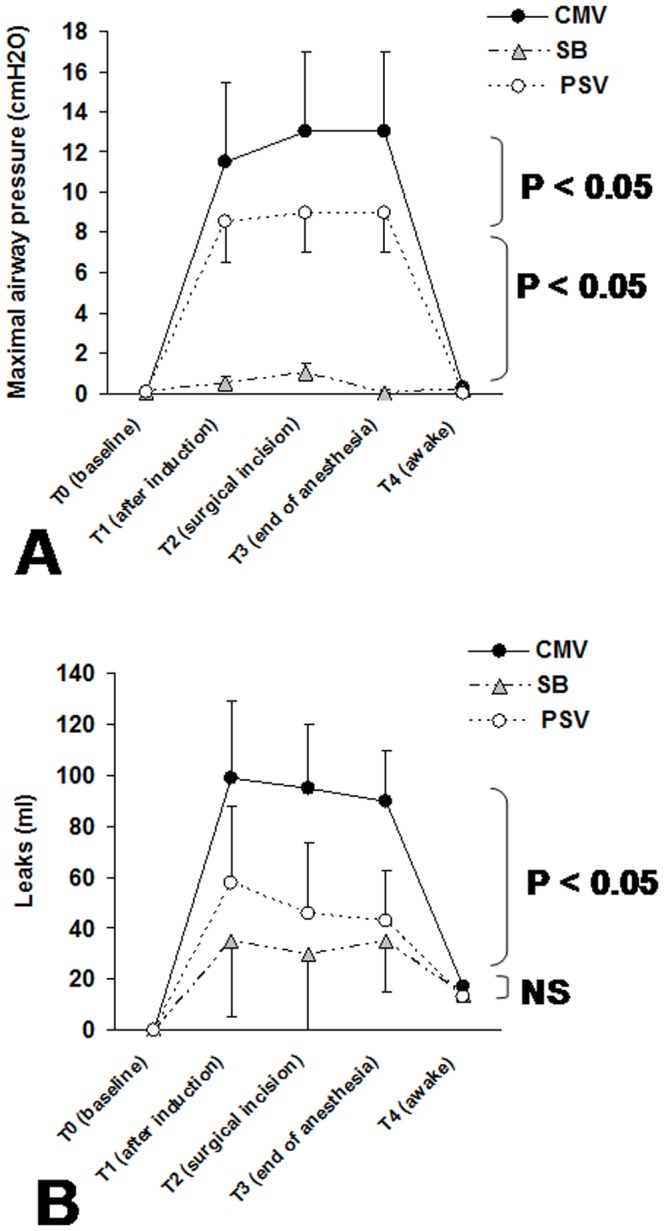
Maximal airway pressure and leaks volume around the LMA. (*A*) Maximal airway pressure and (*B*) leaks volume around the LMA at baseline (T0: before general anesthesia induction), 10 min after general anesthesia induction (T1), at the surgical incision (T2), at the end of anaesthetic drugs infusion (T3) and when the patient was totally awake (T4) obtained for the three groups: CMV-group (Controlled Mechanical Ventilation), SB-group (Spontaneous Breathing) and PSV-group (Pressure Support Ventilation). Data are expressed as mean ± SD. *P* value refers to between-groups comparison. *P<0.05 versus baseline values, NS: not significant.

Fig. 5 shows the maximal airway pressure and leak volume values. For the CMV-group, the significantly higher amount of leaks in comparison to both PSV and SB groups was associated with a significant increase in maximal airway pressure for periods T1, T2 and T3.

## Discussion

Our main results are that intra-operative PSV use during general anaesthesia in unparalyzed patients decreases LMA removal time, emergence time and propofol consumption while improving ventilatory function without adverse effects. All patients tolerated ventilation well. Only one patient in the CMV group displayed methylene blue regurgitation but did not experience complications.

Manufacturers have developed and proposed pressure modes (i.e., pressure-controlled ventilation and PSV) in the newer generations of anaesthesia ventilators [12]. Few studies have evaluated the interest of pressure modes under general anaesthesia in the operating room [11], [15], [16], [25]–[27]. Brimacombe et al. [15] reported that PSV provides more effective gas exchange than does unassisted ventilation with CPAP during anaesthesia with the LMA. In the present study, our results on the breathing pattern during PSV, in comparison to SB, are consistent with those reported in others studies performed in operating room with ICU ventilators [11], [28], [29]. Indeed, we found that, compared to SB, PSV guarantied an optimal tidal volume. Moreover, during SB, minute ventilation decreased significantly by 43% after general anesthesia induction (Fig. 4). With a PSV mode, minute ventilation increased slightly (Table 2, Fig. 4). In the three groups of patients, RR remained constant (Table 2). During SB, VT decreased significantly by 39% (Table 2) with a Pet_CO2_ increase due to the impact of anaesthetic drugs on respiratory control. However, under PSV and CMV, minute ventilation and Pet_CO2_ remained constant. Assisted ventilation modes (PSV or CMV) compensated the suppressed ventilatory control and increased inspiratory work load.

Although the level of delivered assistance by the ventilator was equivalent both in PSV and CMV, as attested by similar plateau pressures, minute ventilation and Pet_CO2_, the total propofol consumption was significantly lower in PSV-group than in CMV-group (Figs. 3–5). PSV has been widely used as a partial ventilatory support for better patient-ventilator synchrony as compared with synchronized intermittent mandatory ventilation or assist-control ventilation [6], [30]. The efficacy of PSV depends on a breath-by-breath interaction between the patient's demand for spontaneous flow and the ventilator's flow. The lower propofol consumption in PSV in the present study may be explained by a better patient-ventilator interaction in PSV mode than during CMV [4]. Indeed, during CMV the patient-ventilator asynchrony (often called “fighting the ventilator”) causes an increase in the need for anaesthetic or sedative drugs infusion [31]. Then, this observed lower propofol consumption in the PSV group could explain the lower general anesthesia emergence time compared to CMV group. Although the P0.1 values obtained in PSV and SB were low (less than 3 cmH2O), the PSV-group had significantly lower P0.1 than in the SB-group suggesting that PSV allowed a decrease in work of breathing in comparison to SB (Table 2). It was reported that spontaneous breathing through the ventilator circuit adds extra workload to the patient as previously reported in critically ill patients [32] or in healthy patients under general anaesthesia [5]. Then, in comparison to SB, PSV permitted to compensated the extra workload due to the ventilator, particularly because the decelerating flow form which permitted to deliver a high peak flow, even with a relatively short inspiratory time [5], [7], [20]. Indeed, in volume modes where the delivered flow is square and often initially automatically set and in patients with spontaneous breathing, the patient's peak flow demand may be higher than the peak flow delivered by the ventilator and thus induce a patient-machine dysynchrony [31].

Gas leak fraction around the LMA was significantly higher in the CMV-group than SB and PSV groups (Fig. 5B). This was due to significantly higher maximal pressure in CMV than in PSV (Fig. 5A). These results are consistent with previous studies which reported that during general anaesthesia with LMA, leaks are significantly higher during CMV or during high maximal airway pressure ventilation in paralysed anesthetized patients [33], [34].

Our study has some limitations. The study was unblinded during surgery for the respiratory and hemodynamic variables. However, at the end of surgery, end points assessors were unaware of the assignment of the group throughout the study. We did not monitor the depth of anaesthesia using the bispectral index (BIS) to ensure that the three groups had a similar level of anaesthesia, but for the three groups we used the usual hemodynamic variables to guide the anaesthetic drugs administration. On the other hand the use of BIS monitoring deserves comments in unparalyzed patients [35]. These results are applicable only for general anesthesia performed with the anesthestic drugs used in the present study (i.e, propofol and fentanyl) and the use of a LMA. Also, we did not record gas exchange, number of asynchronies and work of breathing, but we used the P0.1 as a surrogate for inspiratory effort. Finally, these results were obtained in selected population of young patients with ASA status I and II scheduled for elective knee arthroscopic surgery.

In conclusion, our study suggests that intra-operative PSV in patients with LMA reduces anaesthesia emergence time and propofol consumption compared to CMV. PSV also improves ventilatory function without adverse effects compared to SB during elective surgery under general anaesthesia with a laryngeal mask airway.

## Supporting Information

S1 Protocol **Trial Protocol.**
(DOC)

S1 Checklist **CONSORT Checklist.**
(DOCX)
