# Supplementary material for: Effects of Pressure Support Ventilation Mode on Emergence Time and Intra-Operative Ventilatory Function: A Randomized Controlled Trial
Source: PLoS One. 2014 Dec 23;9(12):e115139. doi: 10.1371/journal.pone.0115139 (PMC4275214; doi:10.1371/journal.pone.0115139)
Supplement: S1 Protocol — Trial Protocol. (DOC) [file pone.0115139.s001.doc]

Protocol S1

**Title:**

**Effects of intra-operative Pressure Support Ventilation mode during general anesthesia:**

**A randomized Controlled Study**

**Xavier. Capdevila^1^, Samir. Jaber^2^**

1. Anaesthesia and Critical Care Department A, Lapeyronie Teaching Hospital, Centre Hospitalier Universitaire Montpellier, Unité INSERM U1046, Montpellier, 34295 France

2. Anaesthesiology and Intensive Care; Anaesthesia and Critical Care Department B, Saint Eloi Teaching Hospital, Unité INSERM U1046, Université Montpellier 1, Centre Hospitalier Universitaire Montpellier, Montpellier, 34295 France

**Head of department when the work should be done** = Pr. Xavier. Capdevila.

Anaesthesia and Critical Care Department A, DAR A.Lapeyronie Teaching Hospital, Centre Hospitalier Universitaire Montpellier, Montpellier, 34295 France

ISRCTN17382426 - <http://www.controlled-trials.com/ISRCTN17382426>

**Key words:** mechanical ventilation, pressure support ventilation, general anesthesia, protective ventilation, surgery; postoperative complications, extubation

E-mails:

**Xavier Capdevila : x-capdevila@chu-montpellier.fr**

**Effects of intra-operative Pressure Support Ventilation during general anesthesia: A randomized Controlled Study**

**PLAN**

1. **Objective**
2. **Hypothesis of the work**
3. **Primary end point**
4. **Secondary end points**
5. **Ethic considerations**
6. **Study design (figure 1)**
7. **Inclusion criteria**
8. **Exclusion criteria**
9. **Randomization procedure**
10. **Premedication protocol anesthesia**
11. **Protocol general anesthesia**
12. **Airway management**
13. **Ventilator and delivered mechanical ventilation**
14. **Respiratory and hemodynamic monitoring**
15. **Statistical analysis**

**Title:**

**Effects of intra-operative Pressure Support Ventilation during general anesthesia: A randomized Controlled Study**

**1. Objective:** Evaluate the safety and efficiency of Pressure Support Ventilation (PSV) mode during general anesthesia in orthopaedic surgery.

**2. Hypothesis of the work:** Pressure Support Ventilation (PSV) allows a reduction of emergence time to extubation, anaesthetic drugs consumption, and improves main ventilatory parameters in comparison to the 2 main standards of care as spontaneous breathing (SB) and controlled mechanical ventilation (CMV).

**3. Primary end point:** The primary end point is LMA removal time with PSV in comparison to CMV. The removal time (open mouth to command) of the LMA will be determined by questioning every minute the patient. The emergence time will be defined as the time to obtain a 10 point score on a five questions test. Each of the following items:1) month of birth; 2) date of surgery; 3) day of the week; 4) address of the patient; 5) simple addition, will scored 0 (no response), 1 (inexplicit response) or 2 (good response).

**4. Secondary end points:**

**-** Total drugs consumption used during TIVA recorded at the end of the surgical procedure.

- Total drugs consumption during TIVA (except induction dose) normalized to weight and duration of TIVA expressed in mg kg-1 min-1 for propofol and µg kg-1 min-1 for fentanyl.

- Hemodynamic (Heart rate, MAP) and ventilatory variables (RR, VTi, VTe, Ti/Ttot, maximal and plateau pressures, SpO2, PetCO2) recorded during a 2-min stable state for each period.

- Peak pressure, plateau pressure and flow will be measured with Capnomac Ultima at the tube opening.

- Airway leaks will be determined by noting the difference between inspired and expired tidal volume (VTi-VTe).

- Airway occlusion pressure (P0.1) will be measured at each point except in T1, T2 and T3 for the CMV group. For T0 and T4, ventilatory variables will be obtained in awake patients spontaneously breathing through the circuit via a mouth piece with a nose clip.

- Presence or absence of methylene blue regurgitation at the end of surgery will be evaluated by an airway fiberoscopy throughout the LMA.

**5. Ethic considerations :** CCPPRB (Comité consultatif de protection des personnes dans la recherche biomédicale) University Hospital of Montpellier, Ethics committee – Montpellier Méditerranée. France 34000.

Informed written consent will be obtained from all patients.

**6. Study design:** prospective randomized control trial including three parallel groups.

All data will be recorded at baseline (T0: before TIVA induction), 10 min after TIVA induction (T1), at the surgical incision (T2), at the end of anaesthetic drugs infusion (T3) and when the patient will be totally awake (T4).

Figure 1 summarizes the study design.

**7. Inclusion criteria:** ASA physical status I and II patients scheduled for elective knee arthroscopic surgery under total intravenous anesthesia (TIVA) with a classic laryngeal mask airway (LMA) and mechanical ventilation.

**8. Exclusion criteria:** emergency procedures**,** ASA physical status > II, refusal patient.

**9. Randomization procedure:** in operating room (OR), the patients will be allocated randomly into three groups (SB, CMV and PSV). The investigators will generated a random-number table on a computer, used the table to prepare envelopes for random patient allocation.

**10. Premedication protocol anesthesia:** oral hydroxyzine (1 mg kg^-1^) and a gelule of methylen blue (2 mg).

**11. Protocol general anesthesia:** TIVA will induced using propofol 2.5 mg kg^-1^, fentanyl 2 µg kg^-1^ and will be maintained with propofol 0.05 mg kg.^-1^ min^-1^ and fentanyl 0.02 µg kg^-1^ min^-1^ with nitrous oxide in oxygen, with a fraction of inspired oxygen of 0.4.

The anaesthetic regimen will be individually adapted to hemodynamics variables according the standard of care of our center. Briefly, additional boluses of propofol 0.25 mg/kg and fentanyl 25 µg will be given in case of a 20 beat min^-1^ increase in heart rate and/or a 25% increase in MAP during 2 min.

**12. Airway management** = LMA (size 4, females; size 5, males) will be inserted according to the manufacturer’s instructions (Laryngeal MaskCo., Henley-on-Thames, UK). The volume of air in the cuff will adjusted at an airway sealing pressure greater than 15 cmH_2_O.

**13. Ventilator and delivered mechanical ventilation** = Mechanical ventilation will be delivered by a Servo 900C ventilator Siemens-Elema, Solna, Sweden which is the only available ventilator with PSV mode in the operating room during the study period. In SB-group, patients will breath spontaneously throughout the ventilator circuit, in CMV-group, patients will be ventilated in CMV (expiratory tidal volume: VTe of 8 mL/kg, a respiratory rate (RR) of 10 cycles/min and an inspiratory/total duty cycle (Ti/Ttot) ratio of 1:3 with a time plateau pressure at 15% of the inspiratory time) and in PSV-group patients will receive PSV (PSV level will be set to obtain a VTe between 7-8 mL/kg and RR between 10-16 cycles/min, inspiratory trigger will be fixed at -2 cmH_2_O and the termination of inspiratory support will be fixed at the drop of flow at 25% peak inspiratory flow value). Ventilation will be performed with zero positive end expiratory pressure in all three groups.

**14. Respiratory and hemodynamic monitoring:** Monitoring will including electrocardiography (ECG), mean arterial blood pressure (MAP) (monitored noninvasively), pulsoxymetry (Sp_O2_) and end expiratory concentrations of oxygen, carbon dioxide (Pet_CO2_), and nitrous oxide (Capnomac Ultima; Datex-Ohmeda, Helsinki, Finland). The Capnomac Ultima (Datex-Ohmeda, Helsinki, Finland) will be used to continuously monitor the airway pressures and flow waveforms which allowed detection of the wasted inspiratory effort on the expiratory part of the flow waveform.

**15. Statistical analysis:**

The primary end point is LMA removal time with PSV in comparison to CMV.

The secondary end points are respiratory variables and total anaesthetic drugs consumption. Previous studies have shown that TIVA emergence time with LMA removal in CMV patients ranges from 15-20 min, with an average of 18 min and an SD of 6 min. We postulate a difference of 50% in the LMA removal time in the PSV group. Therefore, accepting a type I error risk of 5% and a type II error risk of 20% (power 80%), 12 patients would be required in each group to evaluate our hypothesis. Data will be expressed as mean ± SD. Comparison of two means will be performed using the Student t test. Comparison of several means will be performed using repeated-measures analysis of variance and the Newman-Keuls test. *P<*0.05 will considered as significant.
